# Hours of Aggravation

**Published:** 1883-08

**Authors:** W. S. Gee

**Affiliations:** Hyde Park, Ill.


### HOURS OF AGGRAVATION.

W. S. Gee, M. D., Hyde Park, Ill.

The following list of remedies, with the hours of aggravation, has been of use to the writer and may be of interest to others:

12 M. (noon).—Arg. met., Kob., Spig.

1 P. M.—Arg. met., Mag. carb.

2 P. M.—Lach., Calc. carb., Puls., Lob., Nitr. ac., Sang. can., Hell. (2.30).

3 P. M.—Apis, Ars., Asa., Sil., Staph., Sul., Bell.

4 P. M.—Anac., Arum tr., Cal. phos., Carbo veg., Chel., Gels., Hell., Kali hyd., Kob., Lachn., *Lyc.*, Mag. mur., Mur. ac., Nat. sulph., Puls., Stann.

5. P. M.—Actea., Con., Kali c., Puls.

6 P. M.—Bapt., Cal. phos., Caust., Dig., Hepar, Hyp., Kali hyd., Lachn.

7 P. M.—Ant. cr., Bov., Lyc., Pet., Rhus.

8 P. M.—Merc. bij., Tarax.

9 P. M.—Anac., Bry., Mur. ac., Sul. ac.

10 P. M.—Cham., Pod., Puls.

11 P. M.—Bell., Cac. grand., Rumex cr., Sil.

12 P. M.—Acon., Arg. nit., Canth., China, Ferrum, Lachn., Ledum, Mag. c., Mag. mur., Merc. bij., Pet., Pso., Ran. sc., Rhus tox., Sabina, Spong., Staph., Stram., Thuj.

1 A. M.—Ars., Caul., Lachn., Mag. m., Mur. ac., Pso.

2 A. M.—Aurum mur., Cepa, Dros., Ferrum, Kali b., Kali c., Lachn., Mag. c., Rumex cr.

3 A. M.—Am. c., Ant. t., Bapt., Borax, Cal. carb., China, Con., Dulc., Iris v., Kali c., Mag. mur., Nitrum, Nux vom., Pod., Sec. cor., Sepia, Thuj., Zinc.

4 A. M.—Am. c., Caust., Lil. tig., Stan., Nux vom., Kali carb.

5 A. M.—Bov., Helon., Kali hyd., Kob., Pod.

6 A. M.—Aloe, Calc. phos., Ox. ac., Sep., Sil., Sul.

7 A. M.—Eup. perf., Pod.

9 A. M.—Kali c., Pod.

10 A. M.—Actea, Gels., Nat. mur., Rhus, Stan.

11 A. M.—Actea, Arg. met., Ars., Arum. tr., Asa., Berb., Cact. gr., Hydras., Hyos., Ipec., Jacea, Kob., *Sulph.*

